# Acquisition of Lateralized Predation Behavior Associated with Development of Mouth Asymmetry in a Lake Tanganyika Scale-Eating Cichlid Fish

**DOI:** 10.1371/journal.pone.0147476

**Published:** 2016-01-25

**Authors:** Yuichi Takeuchi, Michio Hori, Shinya Tada, Yoichi Oda

**Affiliations:** 1 Department of Anatomy, Graduate School of Medicine and Pharmaceutical Sciences, University of Toyama, Toyama, Japan; 2 Graduate School of Science, Nagoya University, Aichi, Japan; 3 Department of Biological Science, Kyoto University, Kyoto, Japan; 4 Graduate School of Science and Engineering, Ehime University, Ehime, Japan; University of Basel, SWITZERLAND

## Abstract

The scale-eating cichlid *Perissodus microlepis* with asymmetric mouth is an attractive model of behavioral laterality: each adult tears off scales from prey fishes’ left or right flanks according to the direction in which its mouth is skewed. To investigate the development of behavioral laterality and mouth asymmetry, we analyzed stomach contents and lower jaw-bone asymmetry of various-sized *P*. *microlepis* (22≤SL<115mm) sampled in Lake Tanganyika. The shapes of the pored scales found in each specimen’s stomach indicated its attack side preference. Early-juvenile specimens (SL<45mm) feeding mainly on zooplankton exhibited slight but significant mouth asymmetry. As the fish acquired scale-eating (45mm≤SL), attack side preference was gradually strengthened, as was mouth asymmetry. Among size-matched individuals, those with more skewed mouths ate more scales. These findings show that behavioral laterality in scale-eating *P*. *microlepis* is established in association with development of mouth asymmetry which precedes the behavioral acquisition, and that this synergistic interaction between physical and behavioral literalities may contribute to efficient scale-eating.

## Introduction

Behavioral laterality has been reported in a wide variety of vertebrates, including chimpanzees, gorillas, rats, mice, whales, chicks, toads and fish as well as humans [1], and even in crustaceans and insects [2]. The existence of behavioral laterality in lower vertebrates and even in invertebrates implies that it appeared in early Metazoan evolution and that it confers advantages for survival [3]. For example, chimpanzees that specialize at using one hand for termite fishing catch termites more efficiently than individuals lacking handedness [4]. The lateralized pecking behavior of the crossbill is adapted to its skewed mandible (left- or right-directed), which enables it to pick up conifer cones more effectively [5]. Strength, speed, and/or accuracy of movements are generally higher on the dominant side [6, 7]. A growing body of empirical evidence shows that lateralized individuals have advantages over nonlateralized individuals across various types of behavior, and that this phenomenon is taxonomically widespread [8, 9]. Human handedness is believed to be necessary to perform elaborate or dynamic movements (such as writing, throwing, and using scissors), and is known to correlate with the morphological dominance of one arm, expressed in the form of a more massive brachialis muscle or humerus [10, 11].

**Fig 1:**
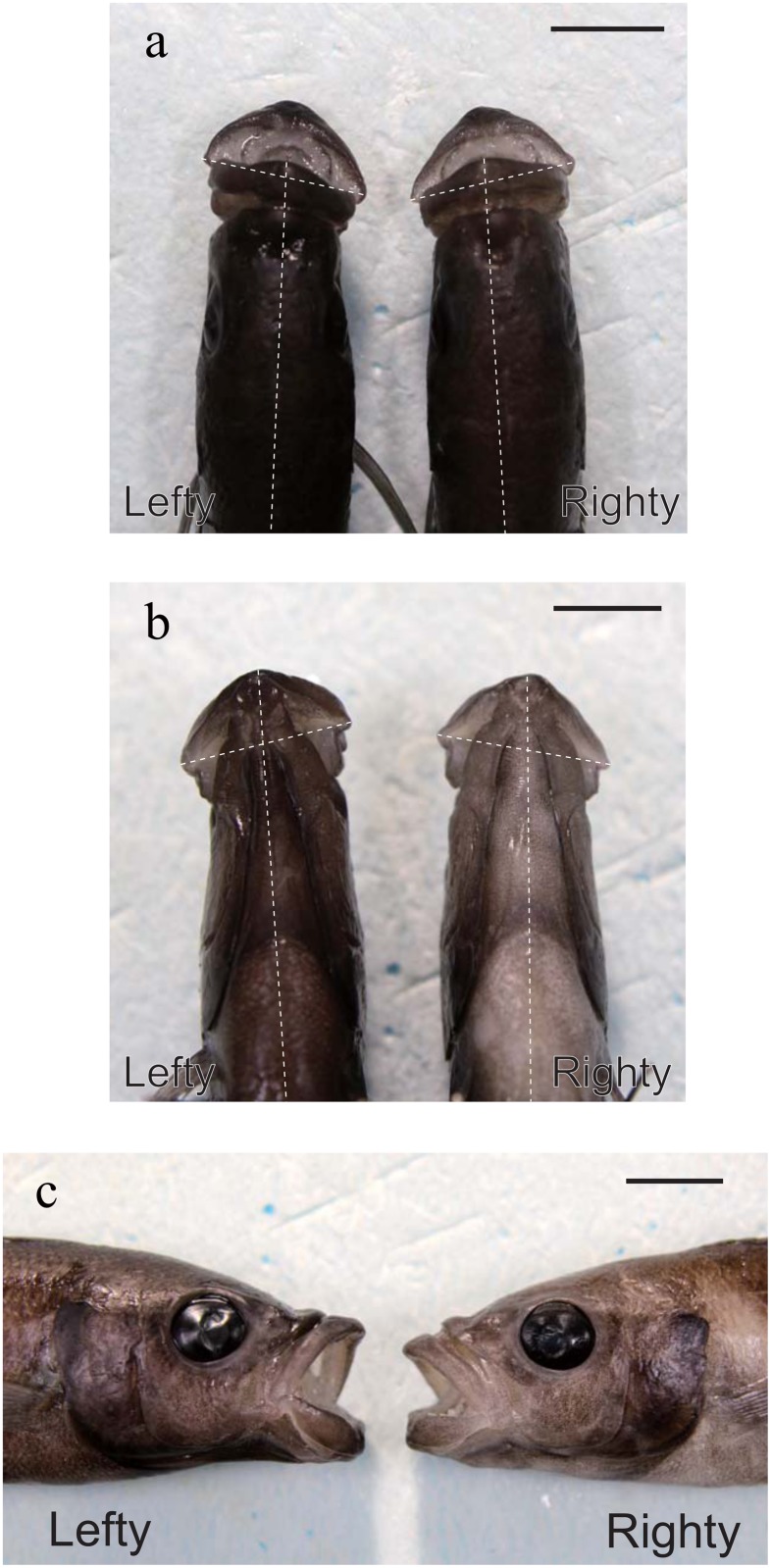
Morphological mouth asymmetry of scale-eating cichlid fish *Perissodus microlepis*. **a**, Dorsal view, **b**, ventral view, **c**, lateral view of the mouth morphologies of lefty and righty fish in adults. The dotted lines indicate the midline and the lateral tips of the lips. In dorsal and ventral views, the line of the lips in the lefty (righty) clearly leans to the right (left). Scale bar = 1cm.

Although phenomenological descriptions of behavioral laterality are abundant, it is not yet fully understood how behavioral laterality is acquired during ontogeny or whether it relates to the inherently asymmetrical processes of brain and visceral organ development. To answer these questions, quantitative analyses must be conducted at various stages of development. In the present study, we employed a simple animal model of behavioural laterality. *Perissodus microlepis*, a scale-eating cichlid fish in Lake Tanganyika, exhibits conspicuously lateralized predation behavior in adulthood. The direction of attack during scale-eating is tightly linked to each individual’s mouth asymmetry (Fig 1), which is skewed either to the left or to the right [12, 13]. Each specimen tears off scales exclusively from one side of its prey fishes’ bodies, as revealed through examination of scales in the stomach contents of the scale-eaters and scars on their prey fishes’ flanks [13] as well as observation of their predation behavior in tanks [14, 15]. About 50% of scale-eaters prefer to attack a prey fish’s left trunk (lefties), while the other 50% prefer to attack the right (righties): lefties have mouths with larger left jaw-bones and preferentially open their mouths to the right to attack the left flank of a prey fish; in righties, these characteristics are reversed [16]. The opening direction of the mouth depends on a one-sided development of the lower jawbone [17,18]. Mouth asymmetry in various fish species including *P*. *microlepis* has been quantified in previous studies in terms of skeletal asymmetry of the lower jaw or the neurocranium–vertebrae angle [15, 16, 18, 19, 20, 21, 22].

This asymmetry of *P*. *microlepis* is presumably advantageous in that it enlarges the contact area between the predator’s teeth and the prey’s trunk [13, 23]. The maximum angular velocity and amplitude of body flexion during a predation attack, as observed in experiments with fish in tanks, is higher when a scale-eater attacks on its preferred side [15]. Previous studies have focused on the ecology and feeding behavior of the adult scale-eater [13, 14, 15, 23]. Their development, however, remains to be examined in depth. It should be noted that mouth asymmetry first appears in the larval stage, though the distribution is unimodal with mean = 0 [24], when the yolk is still present. Scale-eating is thought to begin after the early-juvenile stage in which *P*. *microlepis* forage zooplankton rather than scales [25]. The developmental change in feeding habits, i.e., the transition from eating zooplankton to eating scales, may be related to the accentuation of behavioral laterality and mouth asymmetry.

The aim of the present study is to clarify the development of behavioral laterality in predation and morphological mouth asymmetry in the scale-eater *P*. *microlepis* and the relation between these characteristics. Stomach contents and mouth morphology were analyzed in field-captured scale-eaters ranging from early-stage juveniles to adults. The stomach contents indicated not only the stage of development at which juveniles start foraging scales, but also each specimen’s preferred side of attack, because pored scales from the two sides of a prey fish are mirror-symmetrical in shape. Our results show that the strength of attack-side preference and the degree of mouth asymmetry both increase during development and are positively correlated. Further, we will discuss the evolutional change in left-right mouth asymmetry from the perspective of the relationship between the degree of mouth asymmetry and feeding success.

## Materials and Methods

### Subjects

The scale-eating cichlid *Perissodus microlepis* is widely distributed in Lake Tanganyika [26]. *P*. *microlepis* are specialized to feed predominantly on the scales of prey fishes [27]. In the field, adult scale-eaters prefer to prey on aufwuch feeders, carnivores, and plankton feeders [25]. Early juvenile to adult scale-eaters of both sexes (22–115mm SL) were sampled using a screen net, a hand net and a seine net at the southern end of the lake (Kasenga Point, Zambia; 8°43’S, 31°08’E) in 1990, 1992, 1993, 1996, 2000, 2003, 2011, 2012, and 2014. Immediately after being caught, each scale-eater was put into a small bottle to be euthanized by an overdose of MS-222 (1g/L; Sigma-Aldrich). Euthanasia was achieved within 1 to 2 minutes according to fish body size. To prevent the digestion of food items in the specimens’ stomachs, a small amount of concentrated formalin was injected into the abdomen of each fish. The fish were then preserved in 10% formalin. The standard length (SL) and the sexual gland weight (testis and ovary weight) were measured in order to determine maturity level. Male and female fish were considered sexually mature above the SL of 75mm and 60mm, respectively (S1 Fig). All of the experimental procedures including the method of euthanasia were approved by the Nagoya University Committee on Animal Research (approval number: 14–5) and the experimental methods were carried out in accordance with the approved guidelines. All surgery was performed under MS-222, and all efforts were made to minimize suffering.

### Analysis of stomach contents

To examine the relationship between feeding habits and development of behavioral laterality, we analyzed the stomach contents of fish at various developmental stages (22–115mm SL) under a stereomicroscope. The stomach contents of 235 specimens were quantified by the point method [28] with slight modification as follows (i.e., 4 points for 1/4 fullness, 8 points for 1/2 fullness, 16 points for complete (1×) fullness, and 32 points for twice (2×) fullness). Each specimen’s stomach contents were identified and sorted into eight prey groups as follows: scales, copepods, anabaena, ephemeropteran larvae, trichopteran larvae, fish fry, fish eggs, and unidentified matter. The relative volume of each item to the temporary total points was judged and each item was assigned 0, 0.5, 1, 2, 4, 8, 12, 16, 24, or 32 points. The total number of points finally assigned to each stomach was the sum of the points assigned to each item found in that stomach. Percentages of pooled points (proportions of various diet contents) were calculated for each size class.

### Determination of pored scales’ sides of origin

**Fig 2:**
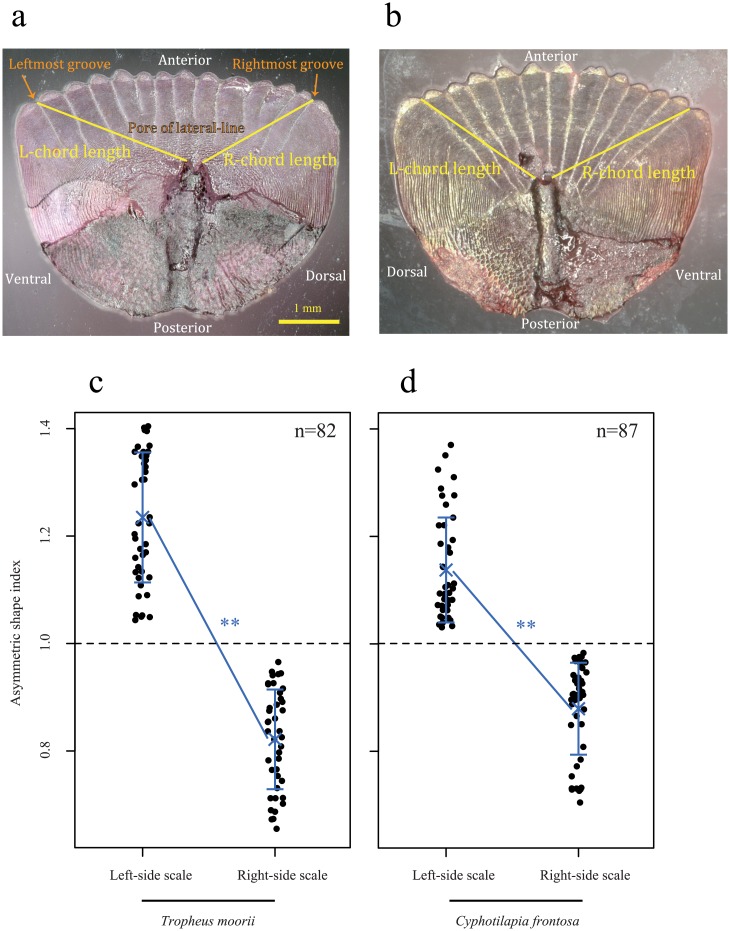
Assessment of behavioral laterality in predation. Pored scales from left (**a**) and right flanks (**b**) of a *Cyphotilapia frontosa*. Ratio of L- to R-chord length (L/R) accurately predicts which flank the scale originated from. The L/R value of left and right side scales in **c**, *T*. *moorii* (82 scales, 58–64mm SL (n = 3)), and **d**, *C*. *frontosa* (87 scales, 75–110mm SL (n = 3)). The cross and bars indicate the mean and standard deviation, respectively. P-values are from the Wilcoxon rank sum test. **P< 0.01.

The pored scales on the left and right flanks of prey cichlids are mirror symmetrical in shape [13]. As scale-eating cichlids swallow whole scales without chewing, the scales found in their stomachs had their original morphology preserved. Therefore, an individual’s preferred attack orientation could be identified from the shapes of the foraged scales in its stomach. We stained pored scales from scale-eater stomachs with alizarin red solution and photographed them using a digital microscope and image analysis software (VHX-2000; Keyence, Osaka, Japan). The length along a lateral line from the pore to the leftmost (L-chord length) or rightmost (R-chord length) constricted part of the groove was measured (Fig 2a and 2b). The value of L/R (called the asymmetric shape index) was used as an indicator of the side of origin of each pored scale. A Wilcoxon rank sum test was performed to test for differences in the asymmetric shape index between the right and left flanks. We confirmed the effectiveness of this method by examining the pored scales of two cichlid species, *Tropheus moorii* and *Cyphotilapia frontosa*, both of which are possible prey species of *P*. *microlepis* in field conditions [25]. For this examination, we used three adult individuals of each species which were obtained from a supplier in Japan (breeding individuals) (*T*. *moorii*: 58–64 mm SL, *C*. *frontosa*: 75–110 mm SL).

### Lateral differences in mouth morphology

**Fig 3:**
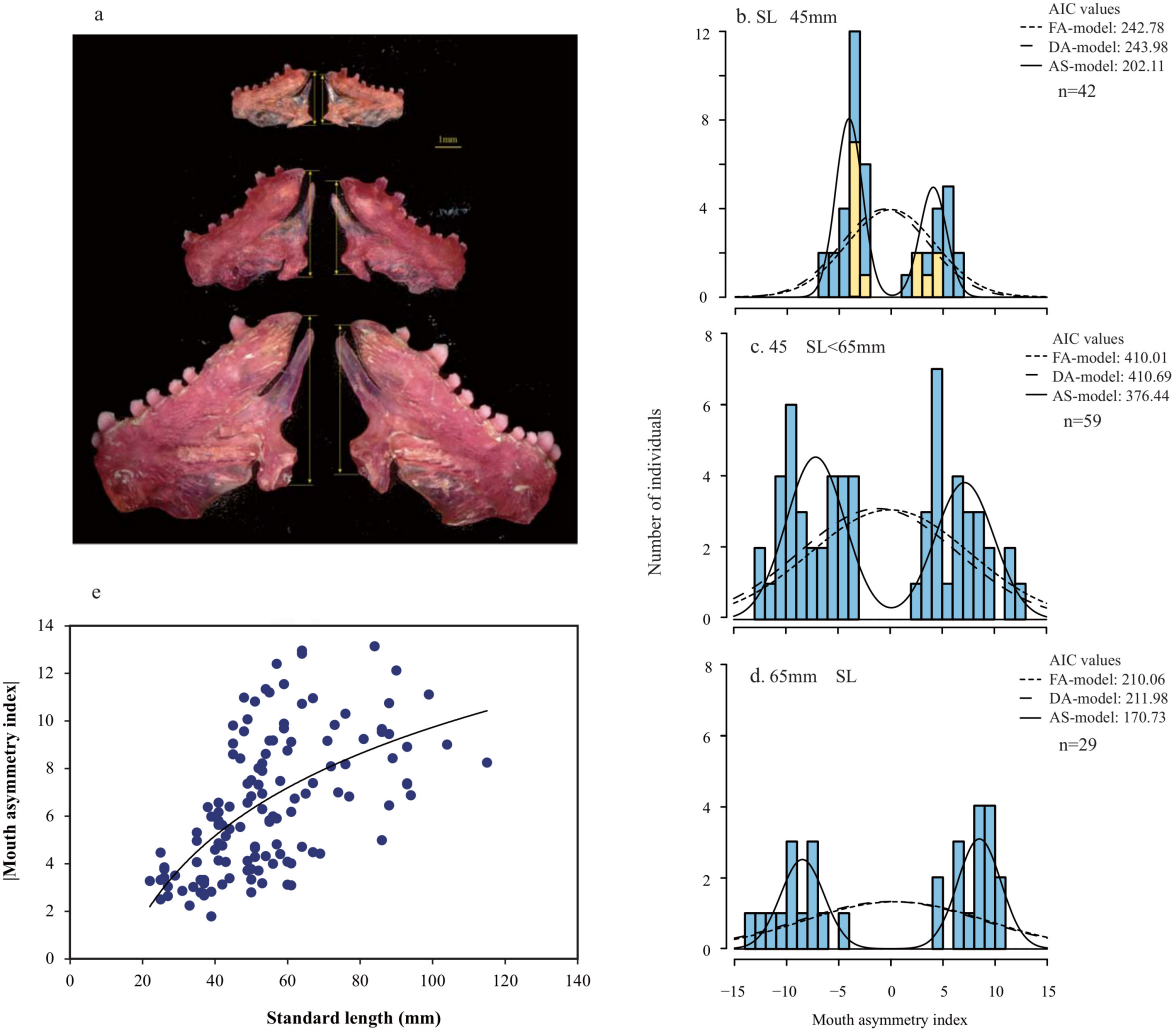
Development of mouth asymmetry in *P*. *microlepis*. **a**, The left and right lower jaw bones of three size classes of lefty *P*. *microlepis* (top: 38mm SL, middle: 64mm SL, bottom: 99mm SL). Arrow length represents the height of the entire posterior dimension of the lower jaw (PD height). The left jaws of each of these individuals were clearly larger than the right jaws. **b, c, d,** Frequency distributions of mouth asymmetry index. **b**, Small-sized fish. The yellow and blue columns represent plankton-feeding fish and omnivore-eating fish, respectively. **c,** Medium-sized fish. **d**, Large-sized fish. The lines indicate the probability curves derived from the three models: the FA-model (dotted line), DA-model (broken line), and AS-model (solid line). **e**, The relationship between body size (standard length) and the absolute value of mouth asymmetry index. The line indicates log-linear regression (y = 4.98ln(x)– 13.17, R^2^ = 0.361, P<0.001).

We measured skeletal features to quantify the asymmetry of the mandibles of larger *P*. *microlepis* specimens that had eaten pored scales and smaller specimens (SL< 45mm) that had foraged on plankton (130 fish). The lower jaw-bones of specimens were carefully denuded of any adhering soft tissue and stained with alizarin red solution. Previously, we had measured the length of the posterior end of each mandible to determine asymmetry [15, 16, 20, 22, 29], but, using this method, it was sometimes difficult to set the criteria points in small fish. As various mouth parts of the scale eater exhibit three-dimensional asymmetry, in the present study, we measured the length of the entire posterior dimension of each lower jaw (referred to as PD height, Fig 3a), rather than the length of the posterior end alone. This measurement method can easily be applied to fish of all sizes, from early juveniles to adults. Lefties had their mouth openings to the right, due to the entire left lower jaw being longer than the right, whereas righties had their mouth openings to the left due to the right jaw being longer [12,13].

We measured the left and right PD heights using a digital microscope. The mandibles were independently positioned on the microscope for each of three replicate measurements to reduce observation error. As the measurement of PD height is prone to yielding some extreme values, median values rather than mean values were used for the following analysis. The measurement errors were negligible small (ANOVA: *F*_129, 650_ = 386.33, P < 0.001). The mouth of each fish opened toward the smaller side of its jaw. The mouth asymmetry index was calculated using the formula [(height R—height L) / (height R + height L) × 2 × 100] [16]. Thus, fish with an index >0 were designated as righties, and those with an index <0 were designated as lefties.

Morphological features can be asymmetrical in three ways, namely, fluctuating asymmetry (FA), directional asymmetry (DA), and antisymmetry (AS) [30]. We performed model selection to determine which of these was most like the asymmetry seen in scale-eater mouth morphology and to generate an index of asymmetry distribution (Package “IASD” in R, detailed in Hata et al. [29]). The FA-model assumes that the data have a normal distribution with a mean of zero (μ = 0) and a standard deviation (± SD), whereas the DA-model assumes a non-zero mean (μ≠ 0) ± SD. The AS-model is based on a bimodal distribution composed of an unequally weighted mixture of two normal distributions with means ± μ (≠ 0) ± SD. In these three models, μ, SD, and fraction of righties or lefties were calculated using maximum likelihood estimation. We applied Akaike’s Information Criteria (AIC) to each of the three models (FA-model, DA-model, and AS-model) to identify the model with the lowest AIC, which is thus the best-fitting distribution type. Moreover, the deviation of each distribution from normal distribution was examined using the Shapiro–Wilk test. Log-linear regression analyses were carried out to test whether mouth asymmetry increases with body size. In addition, to examine the differences in mouth asymmetry between plankton feeders and omnivores among the small fish, a generalized linear model (GLM) analysis was conducted (dependent factors: feeding habit and SL).

### Attack preference for a particular side

The sides of origin of the pored scales found in the scale-eaters’ stomachs were determined based on the asymmetric shape index. This assessment was made only for undigested (undamaged) scales. To assess correlation between the attack side preference and mouth shape asymmetry, we calculated the match ratio, which is the proportion of foraged scales originating from the side of the prey fish corresponding to the direction of the scale-eater’s mouth asymmetry in each individual. For example, a match ratio of 100 percent would mean that a lefty scale-eater had foraged pored scales from the left sides of prey fish only. A generalized linear mixed-model (GLMM) analysis was performed to ascertain the relationship between the degree of mouth asymmetry and the laterality of predation behavior for each individual. Individuals with few pored scales in their stomachs (total number of pored scales <5) were omitted from the analysis. We designed a GLMM with correspondence between mouth asymmetry direction and side of origin of pored scales in the stomach (match: lefty fish fed on left-side scales and righty fish fed on right-side scales; or mismatch: lefties fed on right-side scales and righties fed on left-side scales) as the dependent variable and the following as independent variables: the absolute value of mouth asymmetry index as the fixed effect and individual as the random effect.

To examine the relationship between laterality of predation behavior and body size, an additional GLMM analysis was performed. The dependent variable in our GLMM was correspondence between the direction of mouth asymmetry on the scale-eater and the side of origin of pored scales in its stomach (match or mismatch). The independent variables were body size (SL) as a fixed effect and individual as a random effect.

### Effective morphologicalcharacteristics for scale-feeding success

Feeding success was quantified according to the number of scales in a scale-eater’s stomach. To assess which morphological characteristics may have contributed to their successful scale feeding, a GLM analysis was performed. The number of scales was defined as the dependent variable, whereas standard length and the absolute value of mouth asymmetry index were defined as the independent variables. A GLMM analysis and model selection of frequency distribution were performed using the R statistical software package. Other statistical analyses were performed using JMP version 11 (SAS Institute Inc., Cary, NC, USA).

## Results

### Stomach contents of scale-eaters

**Fig 4:**
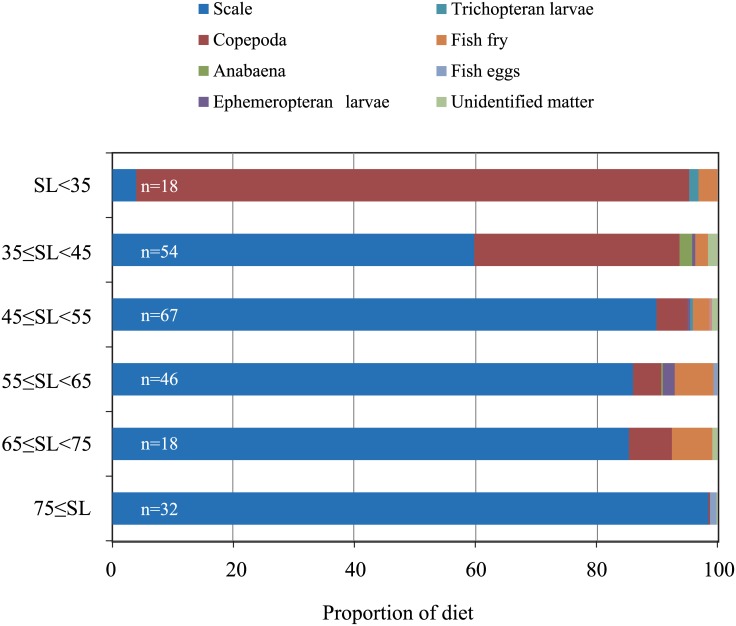
Composition of stomach contents in each size class of *P*. *microlepis*. The stomach contents were classified into eight groups: scales, copepods, anabaena, ephemeropteran larvae, trichopteran larvae, fish fry, fish eggs, and unidentified matter. Prey-item composition is represented as the ratio of each food item to the total points for items found in the stomach by the point method (see Methods). The proportion of scales in the diet drastically increased with body size. n: number of fish; SL: the standard length of fish.

We investigated the development of feeding habits including attack side preference in the scale-eater *P*. *microlepis* by analyzing the stomach contents of specimens with standard length (SL) of body size ranging from 22 to 115mm. Most diet components in the stomachs were identifiable owing to full fixation of the specimens soon after capture. Each specimen was classified as early juvenile, developed juvenile or adult/adult-like (denoted here as “adult”) according to its body size and the maturity of its sexual gland (S1 Fig). In medium- (45≤SL<65, n = 113) and large-sized (65≤SL, n = 50) *P*. *microlepis*, denoted as developed juveniles and adults, respectively, scales of prey fish accounted for more than 80% of the stomach contents (Fig 4). The fraction of scales in the stomach contents was smaller in small fish (SL<45mm, n = 72), denoted as early juveniles; in their place, a higher proportion of copepods was observed along with occasional fish fry or anabaena. Notably, thirteen of these small specimens contained only copepods and were regarded as plankton feeders. The stage-dependent differences in *P*. *microlepis* stomach contents indicates that these cichlids change their primary food source from plankton and omnivores to fish scales as they grow larger than 45mm in SL.

### Asymmetry of scales with lateral-line canals

We examined the shapes of the lateral-line pored scales found in the stomachs of variously sized *P*. *microlepis* specimens to determine which side of the prey fishes’ bodies each scale-eater had preferentially attacked. This analysis was based on the mirror symmetry of lateral-line pored scales from the left and right flanks, as shown previously in scales found in the stomachs of adult *P*. *microlepis*. To confirm the mirror symmetry of lateral-line pored scales of prey fish, we examined the scales from two possible prey fish species (Fig 2a and 2b), one small- and one large-sized (*Tropheus moorii* and *Cyphotilapia frontosa*, respectively). As shown in Fig 2c and 2d, lateral-line pored scales from the left and right flanks were significantly asymmetrical, as represented by the ratio of L-chord to R-chord length in their dorsoventral morphology regardless of the size of the scales or that of the prey fish they came from (Wilcoxon rank sum test: *T*. *moorii*, χ^2^ = 61.50, P<0.001; *C*. *frontosa*, χ^2^ = 65.26, P<0.001). In other words, the scales on the left side of a prey fish’s body are mirror-symmetrical to those on the right side. Therefore, we were able to determine reliably whether each scale had been removed from the left or the right side of the body.

### Lateral asymmetry of lower jaw-bones

The direction of the mouth opening of an individual *P*. *microlepis* is determined by the lateral asymmetry of the lower jaw [16] and is presumed to correlate with the direction of attack during scale-eating behavior. Each scale-eater’s mouth asymmetry was quantified as the bilateral difference in height between the lower jaw-bones ([height R–height L] / [height R + height L] × 2 × 100). Frequency distributions of the mouth asymmetry index revealed a clear bimodal distribution throughout all developmental stages, fitting best to an AS model as determined by AIC model selection because the AIC of the AS model was remarkably lower than those of the FA and DA models. In addition, the distribution deviated significantly from a unimodal distribution (Fig 3b–3d, Shapiro-Wilk test, small fish (SL<45mm): W = 0.838, P<0.001; medium fish (45≤SL<65mm): W = 0.911, P<0.001; large fish (65mm≤SL): W = 0.818, P<0.001). The field-caught population that we studied over a period of eight years contained roughly equal proportions of righty and lefty fish.

There is a significant correlation between body size and the absolute mouth asymmetry index (Fig 3e): both increase more than five-fold from the early juvenile stage to adulthood. Thus, morphological mouth asymmetry increases as fish grow. Among the small fish, the degree of mouth asymmetry did not differ between plankton feeders and omnivore eaters (GLM analysis, df = 2, F = 5.813, P = 0.006; feeding habitat: β = -0.204, SE = 0.293, t = -0.70, P = 0.489, standard length: β = 0.072, SE = 0.042, t = 1.71, P = 0.096). It should be noted that variance in the degree of mouth asymmetry also becomes significantly greater as fish grow (S2 Fig, two-sided F test: F_28, 41_ = 2.652, P = 0.004; Bartlett test: F = 7.972, P = 0.005).

### Establishment of lateralized predation behavior during development

**Fig 5:**
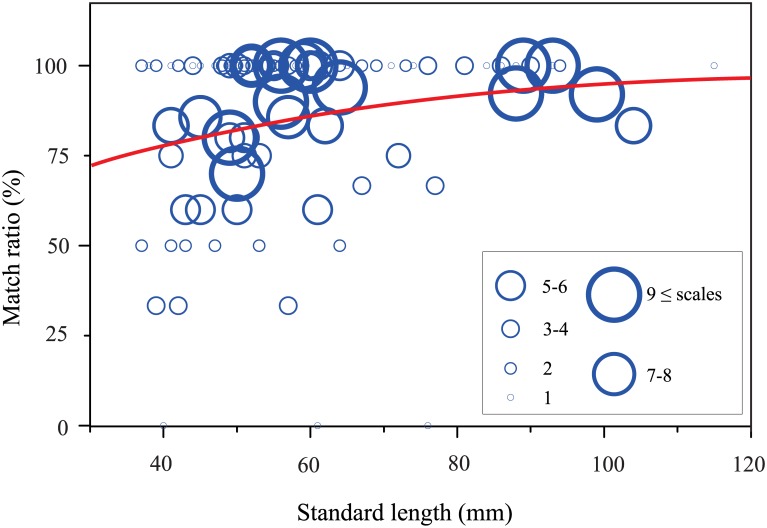
The relationship between body size and match ratio related to behavioral laterality of predation. Match ratio is the percentage of foraged scales robbed from the side of the prey fish that corresponds to the direction of the scale-eater’s mouth opening. The size of each circle represents the number of pored scales in the stomach of an individual fish, as shown in inset. The line indicates the intensity of behavioral laterality in predation predicted by the GLMM analysis (coefficient = 0.027, SE = 0.010, z = 2.447, P = 0.014). Estimated equation: *P* = exp (β_0_ + β_1_*x*) / [1 + exp (β_0_ + β_1_*x*)]. *x*, standard length; β_0_, intercept; β_1_, regression coefficient; β_0_ = 0.462, β_1_ = 0.027.

We next examined the relation between lateralized scale-eating behavior and mouth asymmetry during development. First we found that the distribution of the asymmetric shape index of the pored scales found in the 114 *P*. *microlepis* stomachs examined was bimodal (S3 Fig), as expected based on our examination of scales from possible prey species. Second, the match ratio, that is, the rate of correspondence between the scales’ side of origin and the direction in which the scale-eater’s mouth opened, increased as fish grew (Fig 5; GLMM analysis, coefficient = 0.027, SE = 0.010, z = 2.447, P = 0.014): while the stomach contents of early juveniles (SL<45mm) included scales from both sides, the foraged scales found in adults approaching 80mm in SL were almost exclusively from the preferred side of the prey fish’s flank. Further, the variance of match ratio appeared to decrease in fish over 50mm in SL. There was a significant correlation between mouth asymmetry and the match ratio (GLMM analysis, coefficient = 0.150, SE = 0.071, z = 2.108, P = 0.035). These results indicate that *P*. *microlepis* improves its scale-eating ability by using its skewed mouth more and more effectively as development progresses.

**Fig 6:**
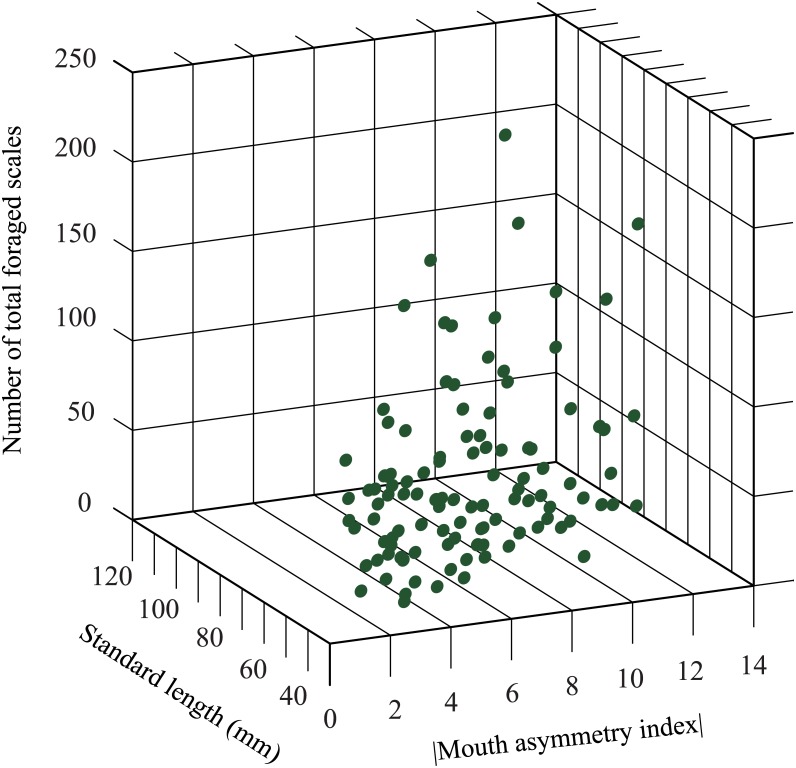
The number of total foraged scales in relation to body size and the absolute value of mouth asymmetry index. Number of foraged scales of each specimen was significantly correlated with both body size and mouth asymmetry (GLM analysis, df = 2, F = 14.657, P<0.001; standard length: β = 0.648, SE = 0.217, t = 2.99, P = 0.003; mouth asymmetry: β = 3.710, SE = 1.331, t = 2.79, P = 0.006).

The relations among the quantity of foraged scales, mouth asymmetry and body size of each specimen are summarized in Fig 6. An individual scale-eater’s success at feeding scales, as indicated by the total number of foraged scales in its stomach, was closely correlated with both its body size and its mouth asymmetry index (GLM analysis, df = 2, F = 14.657, P<0.001; standard length: β = 0.648, SE = 0.217, t = 2.99, P = 0.003; mouth asymmetry: β = 3.710, SE = 1.331, t = 2.79, P = 0.006). A larger fraction of the stomach contents consisted of scales in larger fish. Further, among similar-sized fish, scale-eaters with more dramatically skewed jaws had more scales in their stomachs (see also S4 Fig). Specimens with mouth asymmetry indices exceeding 8 were particularly likely to have consumed large numbers of scales. On the other hands, the mean size of foraged pored scales (L-chord length + R-chord length) correlated with the degree of behavioral laterality (ANOVA; F = 0.004, p = 0.947) in individuals with high foraging motivation (total number of pored scales ≥5, n = 28).

## Discussion

Observing the stomach contents and mouth morphologies of a field-collected population of the scale-eater *P*. *microlepis* has allowed us to investigate the process by which behavioral laterality in scale-eating develops and the correlation between behavioral laterality and mouth asymmetry. Adults exceeding 75mm in SL fed exclusively on scales of prey fish. For the most part, the pored scales in each scale-eater’s stomach came predominantly from one side of the prey fishes’ bodies, specifically, the side corresponding to the direction of the scale-eater’s skewed mouth opening. In other words, lefty (righty) predators disproportionately preyed on the left (right) flanks of their prey fish. These findings confirm those of a previous field study [13]. In contrast, the stomach contents of early-stage juveniles (SL < 45mm) contained scales from both sides of the prey fish, indicating that these scale-eaters attack both sides of their prey at this early developmental stage, then become increasingly specialized at attacking one side as they grow. This is the first demonstration that the behavioral laterality of the scale-eater emerges in association with its body development. The gradual acquisition of lateralized behavior implies that these scale-eaters may have to learn, over time during development, which side allows them to tear off scales more effectively [15].

Relying on field observations alone, it would have been difficult to investigate the behavior of *P*. *microlepis* continuously during development in order to note developmental changes in feeding habits. Instead, in the present study, the acquisition of scale-eating and that of behavioral laterality in predation were assessed based on stomach contents of individuals ranging from the early-juvenile stage to adulthood. The asymmetric morphology of the pored scales from the left and right sides of Tanganyikan cichlid fish enabled us to determine the side of the prey fish from which each foraged scale had been robbed [13]. *P*. *microlepis* voraciously attacked numerous species in the field. It was reported previously that *P*. *microlepis* feed on the scales many (if not all) co-occurring fish species [25]. Here, we confirmed that the pored scales of *Tropheus moorii* and *Cyphotilapia frontosa*, both Cichlidae species that serve as prey for *P*. *microlepis* in Lake Tanganyika, showed obvious lateral differences in shape, regardless of species or body size (Fig 2c and 2d). In contrast, the flank scales of *Lamprichtys tanganicanus* (Poeciliidae), another prey species, were not pored and were accordingly discarded in the present analysis. The pored scales found in the predators’ stomachs are a key indicator of the preferred side of attack, but stomach contents might overestimate the degree of behavioral laterality because they reflect only the result of predation success and not that of failure. If failure occurs more frequently on the non-preferred side, the attack side preference may actually be weaker than that estimated here from the stomach contents. Nevertheless, the stomach contents of juvenile scale-eaters clearly showed that they attacked both sides of their prey with at least some success on each side. In addition, the match ratio, that is, the proportion of the pored scales in a scale-eater’s stomach that were foraged in an attack from that predator’s dominant side, increases with body size. These results strongly suggest that behavioral laterality in predation is strengthened as the scale-eater grows.

Disruptive selection, in which an intermediate phenotype is assigned the lowest fitness, is thought to play a prominent role in the establishment of intraspecific polymorphism (reviewed by Rueffler et al. [31]). However, there has been little empirical evidence concerning morphological traits (except in sticklebacks [32] and Darwin’s finches [33]). Our results revealed that fish with more strongly skewed mouths got more scales (Fig 6), probably because of the larger area of contact between those predators’ mouths and the flanks of prey fish; furthermore, their greater success at predation would tend to lead to more reproduction as well. In contrast, fish with weakly skewed mouths might obtain less food, therefore growing poorly or failing to survive and having less reproductive success. The present study suggests that disruptive selection plays a role in mouth asymmetry in *P*. *microlepis* based on scale-eating efficiency. Extremely pronounced mouth asymmetry, however, would be disadvantageous with regard to other behaviors such as swimming forward to pursue prey fish. Such counter-selection may suppress the species’ tendency to evolve an excessively asymmetrical mouth.

Mouth asymmetry in scale-eaters has been suggested to be controlled by a simple Mendelian one-locus-two-allele system, in which the lefty is dominant over the righty [13, 16]. Previously, Stewart & Albertson [24] found a significant association between mouth asymmetry and genetic linked markers by linkage analysis. However, the population genetic analysis did not detect an association of a microsatellite locus with mouth asymmetry [14], and variation in jaw-bending can be explained by a certain amount of additive genetic variation according to heritability estimates (*h*^*2*^) based on a quantitative genetic model [34]. Also, mouth phenotype is strongly environmentally influenced under conditions requiring predation [35]. Whether mouth laterality is determined by a single locus two alleles remains to be elucidated. A recent genome-wide association study indicated that the *PC6KS* gene, which regulates the NODAL system, correlates with dominant hand use in humans [36]. It would be worthwhile to investigate the role of the NODAL system in the lateralization of the mandible in scale-eaters.

The clear bimodal distribution of the asymmetry index indicates that the mouth asymmetry of *P*. *microlepis* is three times greater than that seen in non-scale-eating cichlids in Lake Tanganyika (algal feeders [16] and shrimp eaters [20]) with closely related genetic backgrounds. Mouth asymmetry of *P*. *microlepis* was also demonstrated by other morphological measurements, though these measurements showed a unimodal distribution of the asymmetric index [37, 38]. The differences between the distributions may be attributable to differences in the measurements, as our studies have analyzed the skeletal asymmetry of the lower jaw-bones or cranial bones, whereas others have analyzed head asymmetry including the chunky soft lip [29]. Therefore, the evolutionary acquisition of scale-eating habits in *P*. *microlepis* may have accelerated the evolution of their mouth asymmetry. The wide variation in mouth asymmetry observed in adult scale-eaters may reflect a phenotypically plastic response induced by repeated attacks directed to the dominant side, as observed in human athletes (such as tennis or volleyball players) or trained rats, in which physical stress or motor activity enlarges bones or muscles on one side of the body [39, 40, 41, 42]. It has been reported that some teleost fishes have adapted their mouth shapes in response to changes in feeding habits under experimental conditions [35, 43, 44]. Van Dooren et al. [35] pointed out that the extent of mouth asymmetry in *P*. *microlepis* varies with foraging experience. Thus mouth asymmetry might be facilitated by external factors in addition to being determined intrinsically.

Behavioral laterality is believed to be linked to the functional lateralization of neural networks in the brain [1]. Visual information is necessary for each component of the predation behavior of scale-eaters, namely, recognizing a prey fish, pursuing it, moving to the preferred side of its body, and targeting its scales [15]. Thus, visual processing in the optic tectum, a visual center in fish [42], is certainly involved in the control of predation. It should be noted that *P*. *microlepis* use one eye predominantly over the other to target scales on prey fish flanks (unpublished observation). Fast body flexion at the initial phase of attacking prey flanks is thought to be initiated by hindbrain reticulospinal neurons involving the paired giant Mauthner cells (M-cell), which receive visual input through the optic tectum [45, 46, 47, 48]. The involvement of M-cells is suggested by the observation that the outward appearance and kinetics of the body bend assumed during an attack on a prey flank closely resemble those of the M-cell-initiated C-shaped bend (C-bend) assumed during fast escape behavior in response to abrupt auditory or visual stimuli. Therefore, the efficiency of signal transmission along the visuomotor pathway, predominantly along the opto-tectal or tecto-hindbrain pathways, may be lateralized, as suggested previously [15]. It has been established, especially in mammals and birds, that experience at early stages of development can modify or remodel the brain circuits and their functions (reviewed by Hensch [49] and Marler [50]); the same is likely true in fish [51, 52, 53]. Thus, early experience of scale-eating in juveniles may contribute to the induction of brain lateralization.

Behavioral laterality in scale-eaters appears to share several features in common with the lateral behaviors observed in wide variety of animals, including handedness in humans. First, lefties and righties coexist within a single population of *P*. *microlepis*, although they represent opposite behavioral preferences. Like human handedness, then, behavioral laterality in scale-eaters is subject to selection pressure for lateral dimorphism rather than uniformity. Second, complicated tasks such as scale-eating are generally performed more effectively when performed on the dominant side [54]. Third, behavioral laterality reflects morphological asymmetry [10, 11]. Fourth, behavioral laterality is reinforced during development [55, 56]. Unlike human handedness, however, behavioral laterality in scale-eaters is observed to increase both qualitatively and quantitatively throughout development, and the genetic system responsible for laterality can be identified when scale-eaters breed in the laboratory. Taken together, the present findings in scale-eaters significantly advance our understanding of the mechanisms underlying behavioral laterality in animals.

## Supporting Information

S1 Fig The correlation between body size (standard length) and sexual gland weight (testis and ovary weight) of *Perissodus microlepis*. **a**, male (n = 181), **b**, female (n = 53). The testis gland in one male specimen could not be measured due to the dehydrated condition of the sample.
(EPS)

S2 Fig Comparison of absolute value of mouth asymmetry in juveniles and adults. The variance of mouth asymmetry was statistically significantly greater in adults than in juveniles according to the two-sided F test and Bartlett test. **P< 0.01.
(EPS)

S3 Fig The frequency distribution of the asymmetric shape index was used to calculate the proportion of foraged scales from each side of the prey fish (n = 407). (EPS)

S4 Fig The correlation between the degree of mouth asymmetry and the number of total foraged scales. The blue, green, and red circles indicate small fish (SL<45mm), medium fish (45≤SL<65mm), and large fish (65mm≤SL), respectively. More strongly asymmetrical fish experienced significantly stronger disruptive performance than more weakly asymmetrical fish (quadratic regression: r^2^ = 0.149, F_2, 106_ = 9.261, P<0.001; y = 35.872–0.395x+0.356x^2^).
(EPS)
